# Transient immune activation without loss of intraepidermal innervation and associated Schwann cells in patients with complex regional pain syndrome

**DOI:** 10.1186/s12974-023-02969-6

**Published:** 2024-01-17

**Authors:** Beate Hartmannsberger, Sabrina Scriba, Carolina Guidolin, Juliane Becker, Katharina Mehling, Kathrin Doppler, Claudia Sommer, Heike L. Rittner

**Affiliations:** 1 University Hospital Würzburg, Department of Anaesthesiology, Intensive Care, Emergency and Pain Medicine, Centre for Interdisciplinary Pain Medicine, Department of Anaesthesiology, Intensive Care, Emergency and Pain Medicine, University of Würzburg, Oberdürrbacher Strasse 6, 97080 Würzburg, Germany; 2 Department of Neurology, University Hospital Würzburg, 97080 Würzburg, Germany

**Keywords:** Complex regional pain syndrome, IENFD, Nociceptive Schwann cells, Mast cells, Langerhans cells, Tissue resident T cells, Dermal B cells, Skin punch biopsy, Chronic constriction nerve injury

## Abstract

**Background:**

Complex regional pain syndrome (CRPS) develops after injury and is characterized by disproportionate pain, oedema, and functional loss. CRPS has clinical signs of neuropathy as well as neurogenic inflammation. Here, we asked whether skin biopsies could be used to differentiate the contribution of these two systems to ultimately guide therapy. To this end, the cutaneous sensory system including nerve fibres and the recently described nociceptive Schwann cells as well as the cutaneous immune system were analysed.

**Methods:**

We systematically deep-phenotyped CRPS patients and immunolabelled glabrous skin biopsies from the affected ipsilateral and non-affected contralateral finger of 19 acute (< 12 months) and 6 chronic (> 12 months after trauma) CRPS patients as well as 25 sex- and age-matched healthy controls (HC). Murine foot pads harvested one week after sham or chronic constriction injury were immunolabelled to assess intraepidermal Schwann cells.

**Results:**

Intraepidermal Schwann cells were detected in human skin of the finger—but their density was much lower compared to mice. Acute and chronic CRPS patients suffered from moderate to severe CRPS symptoms and corresponding pain. Most patients had CRPS type I in the warm category. Their cutaneous neuroglial complex was completely unaffected despite sensory plus signs, e.g. allodynia and hyperalgesia. Cutaneous innate sentinel immune cells, e.g. mast cells and Langerhans cells, infiltrated or proliferated ipsilaterally independently of each other—but only in acute CRPS. No additional adaptive immune cells, e.g. T cells and plasma cells, infiltrated the skin.

**Conclusions:**

Diagnostic skin punch biopsies could be used to diagnose individual pathophysiology in a very heterogenous disease like acute CRPS to guide tailored treatment in the future. Since numbers of inflammatory cells and pain did not necessarily correlate, more in-depth analysis of individual patients is necessary.

**Supplementary Information:**

The online version contains supplementary material available at 10.1186/s12974-023-02969-6.

## Introduction

Complex regional pain syndrome (CRPS) emerges after trauma or surgery in the affected extremity. A prerequisite for the diagnosis is pain disproportionate to the initial insult. Diagnostic criteria, based on the Budapest criteria, include symptoms such as allodynia or hyperalgesia, oedema, sweating, skin discoloration, temperature changes, motor dysfunction, and trophic alterations. In sensory testing, patients have signs of mechanical hyperalgesia and allodynia as well as cold allodynia [1]. Long-term consequences—debilitating pain and motor impairment of the affected limb—result in inability to work and lowered quality of life of patients [1, 2].

While the underlying aetiology of CRPS is not completely understood, abnormalities in peripheral nociceptive stimulation play a role [1]. Exaggerated post-traumatic inflammation supposably drives CRPS pathogenesis, e.g. substance P- and calcitonin-gene related peptide-mediated neurogenic inflammation as well as autoimmune mechanisms [3–5]. Augmented pro-inflammatory cytokines such as tumour necrosis factor-α (TNF-α) or interleukin-6 (IL-6) as well as autoantibodies arise in CRPS patients which can elicit pro-nociceptive effects in preclinical models [6–10].

First biomarkers have been found in the skin of CRPS patients [4, 11]. In artificial skin blister fluid or skin homogenates, increased inflammation was demonstrated by pro-inflammatory cytokines such as IL-6 or TNF-α [8, 9, 12, 13] and increased mast cell markers [7, 14]. Immunostainings of skin punch biopsies reported similar results as well as changed Langerhans cell densities in different CRPS cohorts [7, 15, 16].

Epidermal and dermal nerve fibres are reduced in the affected limbs of patients with long-standing CRPS [17–20]. Recently, ‘nociceptive’ Schwann cells transmitting noxious stimuli have been found to accompany these fibres across the dermal-epidermal border [21]. Ablation of nociceptive Schwann cells in preclinical models results in small fibre retraction as well as mechanical, cold, and heat hyperalgesia—neuropathic-like symptoms [22]. However, their role in human patients is under debate since its first description. Demise of nociceptive/intraepidermal Schwann cells could contribute to fibre loss and pain generation and/or maintenance in CRPS.

In this study, we aimed to identify factors, such as immune and innervating cells residing in the skin, that might contribute to the pathophysiology in acute CRPS-affected skin. CRPS patients represent a heterogeneous disease group, therefore identification of subtypes driven by specific cell types or mechanism might be revealed by investigating a broad range of targets potentially exhibiting pro-nociceptive function: the glioneural complex (IENFs and Schwann cells) and immune cells (Langerhans, mast, and T cells).

## Materials and methods

### Patient cohort

In this monocentre study at the Centre for Interdisciplinary Pain Medicine (ZiS) at the University Hospital Würzburg, patient recruitment (between 2017 and 2021) followed the *ResolvePAIN* study protocol registered in the German clinical trial register (https://drks.de/—registration number DRKS00016790). Patients were included at their first visit at the ZiS. Ethical approval was obtained from the responsible ethics committees of the University of Würzburg. Study participants were over 18 years old, of both sexes and gave written informed consent. We only included patients with CRPS of the upper extremity. Pre-existing severe neurological diseases, diabetes, current or past malignancies, surgeries within the last 4 weeks at the affected extremity, and current infections were exclusion criteria. As controls, sex- and aged-matched healthy volunteers were recruited for a unilateral finger biopsy.

### Clinical assessment and patient reported outcomes

CRPS diagnosis was based on the Budapest criteria. All patients underwent a clinical neurological examination including the CRPS severity score (CSS). The examination included the reaction to touch, pinprick, pressure, light touch as well as warm and cold stimuli. Skin temperature, swelling, discolouration of the skin, trophic and motor changes were also recorded. For determination of CRPS type I or II, electrophysiological measurements were taken. Patients with a lesion of nerves (nerve conduction velocity and the amplitude of the compound muscle action potential) were classified as type II, otherwise type I.

Numeric rating scale (NRS; 1–10) was used to describe mean pain intensity. For the assessment of neuropathic pain levels, participants filled in the Neuropathic Pain Symptom Inventory (NPSI, German version) which describes the expression of different neuropathic pain characteristics with a score between 0 and 100 [23]*.* Depressive symptoms were measured with the Beck Depression Inventory-II, German version [24]. Scores range from range 0 to 63, scores under 13 are considered mild depression. Participants filled in the State-Trait Anxiety Inventory, German version, (trait anxiety subscale STAI-T) to record anxiety symptoms using a score between 20 and 80 [25]; scores less than 40 were considered normal. To assess functionality of the upper extremity the DASH (Disability of arm, shoulder and hand) score, German version, was recorded [26]. The score ranges from 0 to 100, with 0 indicating full functionality and 100 a complete loss of functionality.

The CRPS diagnosis is usually made up to 6 months after the onset of symptoms [27]. Many of our own patients reported that they knew immediately after the inciting event that something was wrong. Since not all symptoms of the Budapest criteria start at the same time point and recollection of this is notoriously difficult in retrospect, we decided to use the date of the initial trauma or surgery as the onset to define disease duration. This obviously neglects the fact that some patients initially have a normal healing process and CRPS develops weeks after the event.

### Human skin biopsies and plasma/blood collection

Glabrous skin punch biopsies with a diameter of 4 mm were taken from the lateral side of the first proximal phalanx of digitus II at the transition from hairy to glabrous skin. Biopsies were performed at the similar area on a more affected finger (n = 3), when CRPS symptoms were detected at a different site during the clinical assessment. Skin was directly fixed in 4% freshly thawed paraformaldehyde for 30 min, washed in buffer 3 times and cryoprotected in a 10% sucrose and 0.1% sodium azide solution for at least 24 h at 4 °C. Samples were placed in Tissue-Tek® O.C.T. Compound (Sakura Finetek Europe B.V.), frozen in liquid nitrogen-cooled methylpropane and stored at − 40 °C until further use.

### Murine skin samples

All animal experiments were approved by the Government of Lower Franconia, Germany (Regierung von Unterfranken). Male and female C57BL/6JRj mice (Janvier Labs, Le Genest-Saint-Isle, France) were kept under pathogen-free conditions with controlled light cycle, temperature, and humidity (14:10 h light/dark cycle, 20–24 °C, 45–65% humidity). Eight weeks-old mice were provided with standard chow and water with access ad libitum. Chronic constriction injury (CCI) was conducted similarly as previously described [28]. Briefly, mice were deeply anaesthetized with 2–4% isoflurane. The right sciatic nerve was surgically exposed and 3 loose silk ligatures were placed around it with about 1 mm spacing. Ligatures were tied until the nerve was slightly indented. Sham-operated mice had the same surgery without placements of the ligatures.

Seven days after CCI, mice were euthanized and glabrous skin containing foot pads was dissected from the hind paw. Samples were immediately fixed in 4% paraformaldehyde for 1 h, washed in buffer 3 times and cryoprotected in a 10% sucrose and 0.1% sodium azide solution for 24 h at 4 °C. Samples were placed in Tissue-Tek® O.C.T. Compound, frozen in liquid nitrogen-cooled methylpropane and stored at -40 °C until further use.

### Immunofluorescence

We established the markers for intraepidermal Schwann cells based on *Abdo *et al*.* [21]: They used antibodies against S100β and PGP9.5 and genetically induced fluorescent markers for proteolipid protein (PLP)-positive and Sox10-positive cells. Commercially available Sox10 antibodies did not label human or mouse skin (Santa Cruz, #sc365692; Invitrogen, #PA5-40697). PLP immunoreactivity was only detected in larger dermal nerves, but not in the epidermal-dermal region in human and mouse skin (LSBio, #LS-C74986; Novusbio, #NB100-1608; Abcam, #ab28486). Murine nerve fibres could not be stained using different antibodies (anti-NF200, Sigma Aldrich, #N0142; anti-PGP9.5, abcam, #ab10410; anti-PGP9.5, BioRad, #7863–1004; anti-PGP9.5, Merck-Millipore, #AB5898). Since human melanocytes were also positive for S100, melanocytes were counterstained using a specific antibody (1:100, mouse anti-Melan-A antibody, Dako, #M719629). Since melanocytes are morphologically different from Schwann cells and do not have processes, counterstaining was not necessary for our analyses.

Skin samples were cut into 40-µm sections with a Leica CM3050 S cryostat, 10-µm sections were cut for the lymphocyte staining. Three non-consecutive sections were collected on one slide. For mast cell staining, heat-induced antigen retrieval was performed. Sections were boiled in 10 mM citrate buffer, pH 6.0 for 25 min, washed and subsequently stained as follows.

Murine sections were blocked with 5% normal donkey serum in PBS for 1 h at room temperature. Samples were first incubated with rabbit anti-S100 (ready-to-use; Dako #GA50461-2) and goat anti-collagen IV (1:100; Southern Biotech #1340–01) primary antibodies overnight at 4 °C and then incubated with secondary antibodies (anti-rabbit Alexa Fluor 594, Invitrogen, #A21207; anti-goat Alexa Fluor 647, Invitrogen, #A21447) diluted 1:800 in PBS for 1 h at room temperature. Samples were washed 3 times for 30 min in PBS at room temperature to reduce cross reaction with another round of incubation with primary rabbit anti-PGP9.5 (1:200; Zytomed #516–3344) antibody and subsequent secondary antibody (anti-rabbit Alexa Fluor 488, Invitrogen, #A21206). All slides were mounted using a water-soluble mounting medium (Polysciences #18606–20).

Human samples were blocked with 10% bovine serum albumin in phosphate buffered saline (PBS) for 30 min at room temperature. Sections were incubated with primary antibodies overnight at 4 °C: rabbit anti-S100 (Dako), mouse anti-PGP9.5 (1:100; BioRad #7863–1004), rat anti-langerin/CD207 (1:500; Dendritics #DDX0362P-100), goat anti-collagen IV (Southern Biotech), or rabbit anti-C5a (1:100; Bioss #bs-10476R), mouse anti-tryptase (1:1000; Abcam #ab2378), or rat anti-CD3 (1:300; bio-rad #MCA1477), rabbit anti-CD27 (1:300; abcam #ab131254), and mouse anti-CD138 (1:200; bio-rad #MCA2459GA). After washing, samples were incubated with suitable secondary antibodies raised in goat or donkey for 2 h at room temperature: anti-rabbit IgG conjugated with 488- or 594-fluorophore (1:800; Invitrogen, #A21206 or #A21207), anti-mouse IgG conjugated with 488- or 594-fluorophore (1:800; Invitrogen, #A21202 or #A32744), anti-rat IgG conjugated with 405- or 594-fluorophore (1:800; Invitrogen, #A48268 or #A11007), anti-goat IgG conjugated with 647-fluorophore (1:800; Invitrogen, #A21447). For mast cell and lymphocyte staining, nuclei were stained with Hoechst33342 (1µg/ml, Sigma-Aldrich, 14533-100MG).

### Image analysis

Images of the immunofluorescent stainings were acquired using the Zeiss Axio Imager 2, the ZEN3.3 software (Zeiss #410135–1002-330) and saved in *.czi* format for later analysis. Contrast and brightness of recorded images were adjusted immediately after taking the pictures with the function ‘best fit’. For cell counting, the staining was assessed directly through the ocular of the microscope. For each skin sample, the parameters were assessed in 2–3 different sections and averaged for statistical analysis. The following parameters were determined by the same investigator blinded to sample allocation using a 20 × objective: Intraepidermal nerve fibre density (IENFD) and Schwann cell analyses were performed on human and murine samples in the same way. IENFD was determined according to guidelines [29]. Colocalization of PGP9.5 and S100 was interpreted as a nerve fibre and an associating Schwann cell process. Whenever an IENF was accompanied by a signal of S100 this was counted as an intraepidermal Schwann cell. Langerhans cells and Meissner corpuscles were counted under the microscope, papillae later from images taken at a 5 × objective. Data are presented as per mm and Meissner corpuscles per papillae.

For mast cell and C5a analysis, z-stacks of 10 planes and 3 µm step size were acquired covering a total of 27 µm using the same exposure times without contrast adjustment for all samples with a 10 × objective. Each z-stack was maximum projected and further analysed with Fiji ImageJ. Regions of interest (ROIs) for each sample were created manually including the whole dermis with the border of the epidermis. After maximum and top hat filtering of the tryptase channel, a binary image was created and cells larger than 30 pixels were counted as mast cell. The density of mast cells is presented as cells/mm^2^. For C5a analysis, maximum projections of the z-stacks were generated. The ROIs of the mast cell analysis were adjusted, so that the epidermal border was excluded. The mean intensity of each ROI was measured and averaged for analysis.

For lymphocyte analysis, T cells (CD3^+^) and plasma cells (CD27^+^CD138^+^) were counted manually in the dermal and epidermal regions. 20 × tile images of complete skin sections were captured. The cutaneous area (dermis and epidermis) and the epidermal lengths were measured using ImageJ.

### Blood/plasma collection and fluoroenzyme immunoassay

Blood was collected in 9 ml-EDTA tubes (S-Monovette^®^ K3 EDTA, 9 ml; #02.1066.001; Sarstedt AG & Co. KG, Nürnbrecht, Germany) and centrifuged for 10 min at 1300xg at room temperature. The resulting plasma was stored at −80 °C until further analysis. To determine tryptase concentration in plasma, fluoroenzyme immunoassay was performed. The ImmunoCAP™ Phadia™ 250 instrument was used following the manufacturer’s instructions (Thermo Fisher Scientific Inc., Waltham, Massachusetts, USA).

### Statistical analysis

Statistical analysis was done using GraphPad Prism version 9.3.0 for Windows (GraphPad Software, San Diego, USA). Outliers were identified using ROUT methods with Q = 2% and trimmed for subsequent statistical testing. The number of outliers is noted in the corresponding figure legends with ‘#non-outliers \ #outliers’. Shapiro–Wilk test was performed to assess if data were normally distributed. To compare all groups with each other, Welch’s ANOVA test was used when data were normally distributed. For non-parametric data, Kruskal–Wallis test was performed. Dunnett’s or Dunn’s multiple comparisons tests were also performed. Correlation of data was done using Pearson method. Data are shown as mean ± standard deviation (SD) or median with interquartile range. p-values < 0.05 were considered statistically significant.

## Results

### Characteristics of the CRPS patient cohort

Our cohorts consisted of 19 acute (< 12 months since inciting event; 5.2 ± 2.8 months) and 6 chronic (> 12 months; 19 ± 4.8 months) CRPS patients. A summary of demographic and clinical data is presented in Table 1. Although the distributions of age and CRPS type differed between the acute and chronic CRPS groups, CSS and mean pain matched well. Patients suffered from moderate to severe disease symptoms as measured by the CSS and had moderate mean pain.

**Table 1:** Summary of demographic and clinical data of healthy control and CRPS study cohorts

	Acute CRPS n = 19	Chronic CRPS n = 6	HC n = 25
Age (years)	54 ± 9	40 ± 13*	51 ± 11
Sex (female in %)	74%	100%	80%
CRPS type I	58% (n = 11)	83% (n = 5)	
Initially warm	63% (n = 12)	50% (n = 3)	
Time since event (months)	5.2 ± 2.8	19.0 ± 4.8	
Time since diagnosis (months)	2.1 ± 2.5	7.3 ± 8.6	
Mean pain (NRS)	5.3 ± 2.2	5.0 ± 2.2	
Max pain (NRS)	7.4 ± 2.2	7.2 ± 3.0	
Allodynia	37% (n = 7)	17% (n = 1)	
Hyperalgesia	53% (n = 10)	66% (n = 4)	
Oedema	79% (n = 15)	66% (n = 4)	
Skin temp (Δ°C)	-0.3 ± 1.4	0.5 ± 0.9	
CSS^1^	10.4 ± 1.9	10.7 ± 2.1	
NPSI^2^	30.4 ± 22.2 (n = 16)	35.5 ± 28.1	3.2 ± 4.8 (n = 20)
STAI-T^3^	45.0 ± 11.0	38.7 ± 17.3	31.7 ± 7.2 (n = 24)
BDI-II^4^	14.6 ± 10.4 (n = 17)	9.8 ± 9.5	3.9 ± 4 (n = 24)
DASH^5^	62.4 ± 20.9 (n = 18)	37.7 ± 23.1	3.5 ± 4.1 (n = 23)

^1^CRPS severity score, range 0–17

^2^Neuropathic pain symptom inventory, range 0–100

^3^State-Trait anxiety inventory trait anxiety subscale, range 20–80

^4^Beck depression inventory II, range 0–63

^5^Disability of arm, shoulder and hand, range 0–100. CRPS: complex regional pain syndrome; HC: healthy control; *p < 0.05 compared with acute CRPS group

In general, CRPS patients were mostly female (80%) and middle-aged (51 ± 11 years). Almost two thirds of patients showed no nerve lesion (CRPS type I) and an “initially warm” phenotype. At the time of patient examination and biopsy collection, 72% had oedema in the affected extremity and 44% temperature differences between both hands. Pain generally had a neuropathic character and 30% of the patients suffered from allodynia or mechanical hypersensitivity, respectively. Additional file 1: Table S1 presents detailed demographic and clinical data of each CRPS patient.

### Maintained intraepidermal nerve fibre and Schwann cell densities in CRPS

**Fig. 1:**
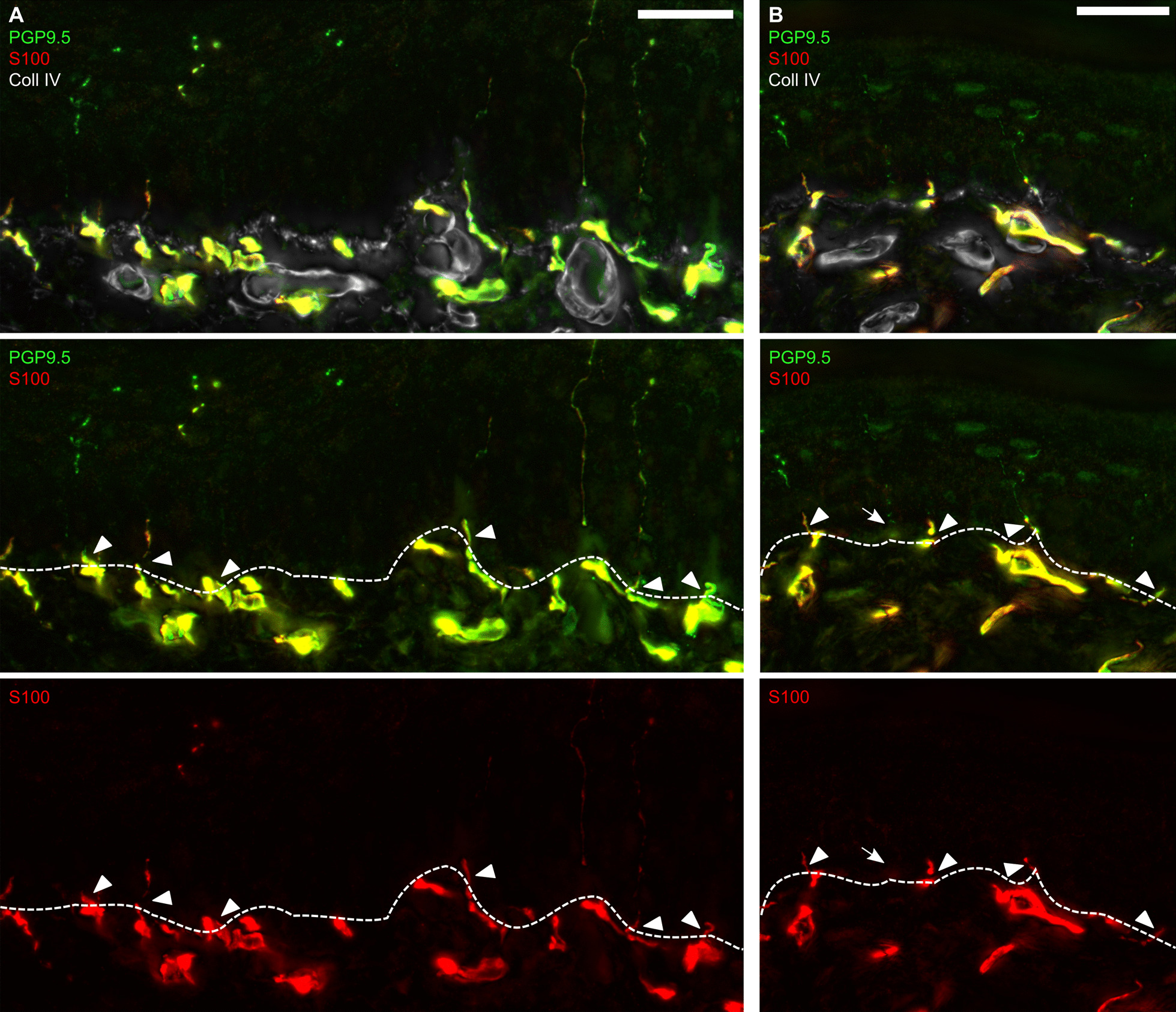
Murine intraepidermal Schwann cells in glabrous plantar skin. **A**, **B** Representative images of immunostainings of skin sections harvested from hind paw foot pads are displayed (n = 2): Schwann cells were labelled with anti-S100 antibody while intraepidermal nerve fibres were identified with anti-PGP9.5 antibody. The epidermal-dermal border was visualized with anti-Coll IV antibody. Fibres and Schwann cell processes crossing the epidermal-dermal border (dashed line) were counted. Arrows indicate IENF without intraepidermal Schwann cell process, arrowheads intraepidermal Schwann cell processes. Scale bar = 25 µm

For this study, we concentrated on intraepidermal Schwann cell processes as S100^+^/CD207^−^ processes that colocalized with PGP9.5^+^ fibre-like structures adapted from the previous studies [21, 22]. We did not assess intraepidermal Schwann cell somata but focused solely on their processes, because we could only rarely detect the somata. To validate our adapted counting method for intraepidermal Schwann cell processes, we first assessed them in mouse plantar hind paw skin 7 days after CCI or sham surgery, since the quantification of intraepidermal Schwann cell processes has been limited to murine tissue so far [22]. Representative images of IENFs with and without intraepidermal Schwann cell processes in mouse are shown in Fig. 1. Counterstaining for CD207 was not necessary since murine Langerhans cells are S100-negative.

**Fig. 2:**
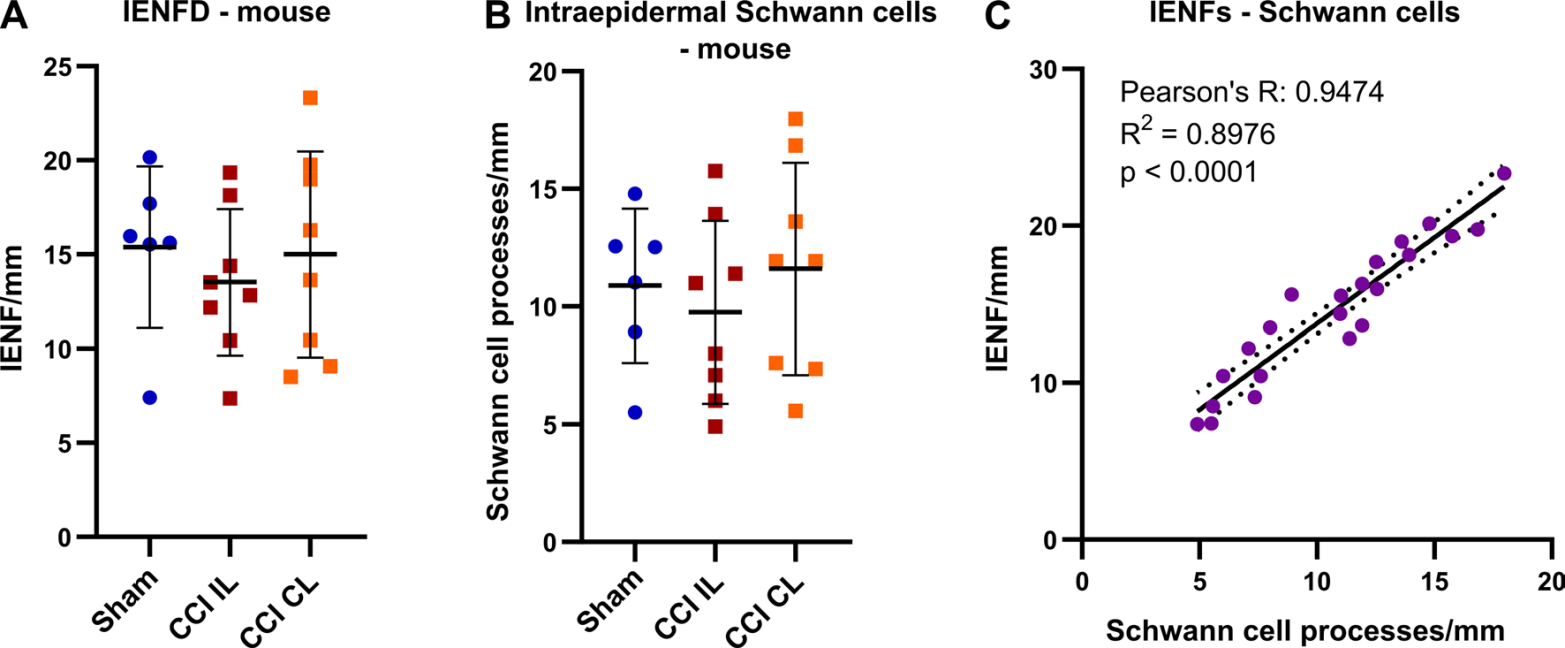
Strong correlation between intraepidermal nerve fibres and associated Schwann cells in murine plantar skin. Male and female mice underwent chronic constriction injury (CCI) or sham surgery. Hind paw foot pads were harvested 7 days after surgery and intraepidermal nerve fibre density (IENFD), and the density of intraepidermal Schwann cell processes were determined. **A** IENFD and **B** associated Schwann cell numbers are plotted (Welch’s ANOVA and Dunnett’s tests, n_Sham_ = 6, n_CCI IL_ = 8, n_CCI CL_ = 8). **C** Pearson correlation between IENFD and Schwann cell process density in all skin samples (n = 22). Data are shown as mean ± SD. CL: contralateral; IL: ipsilateral

IENFD was not changed in mice with CCI injury (Fig. 2A) and varied substantially within each group. Many IENFs were accompanied by intraepidermal Schwann cell processes and their density did not differ between groups (Fig. 2B). IENF and intraepidermal Schwann cell processes correlated strongly (Fig. 2C) supporting the previously reported interdependence of nociceptive Schwann cells and IENFs [22]. Thus, we considered our counting method suitable for further analysis of human tissue.

**Fig. 3:**
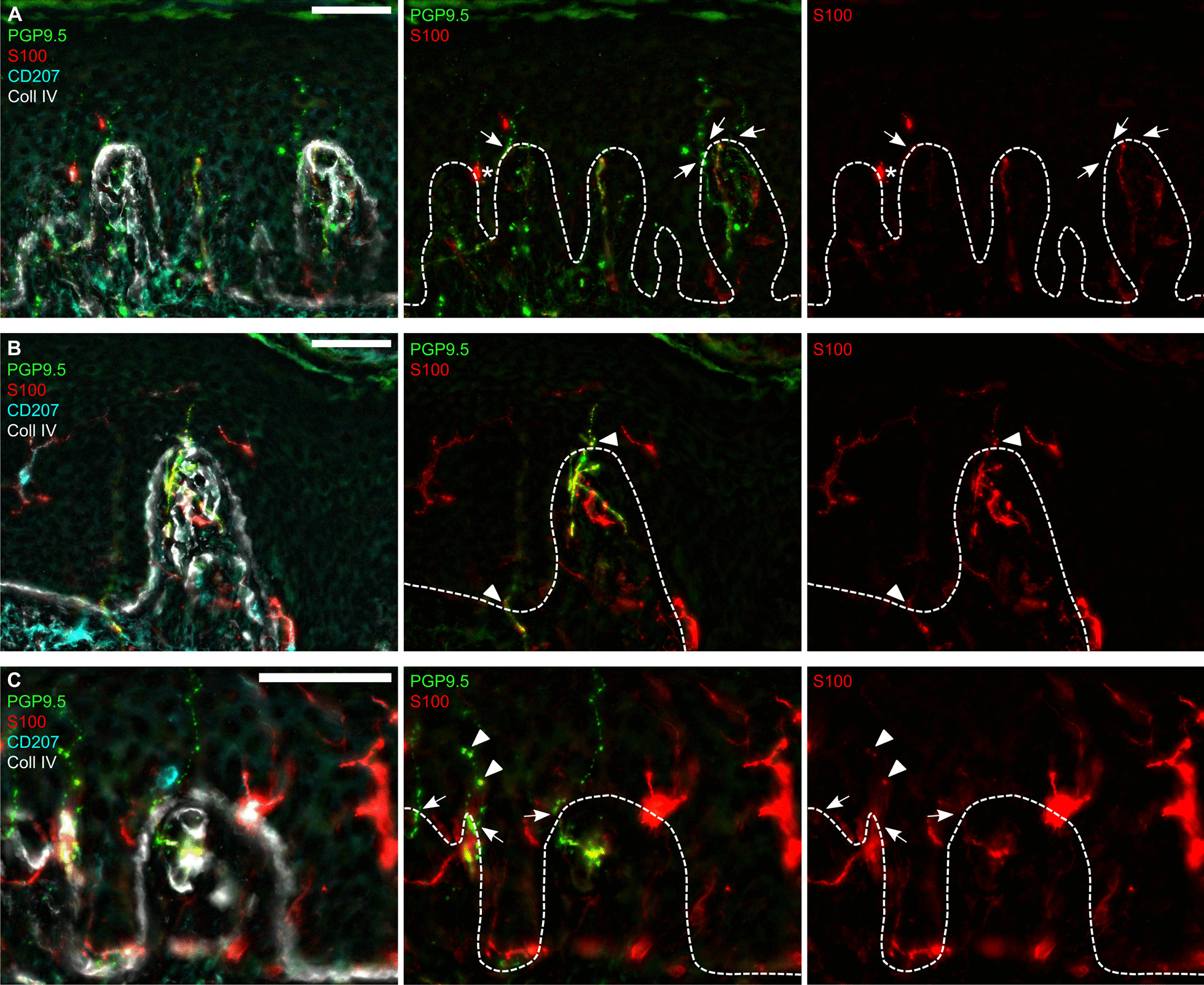
Intraepidermal Schwann cells in human glabrous skin. Skin biopsies from the index finger at the border between glabrous and hairy skin were immunolabelled. **A**, **B**, **C** Representative images of intraepidermal Schwann cell processes (S100^+^), intraepidermal nerve fibres (IENFs, PGP9.5^+^), Langerhans cells (CD207^+^), and the epidermal-dermal border (collagen IV, Coll IV^+^). Nerve fibres crossing the epidermal-dermal border (dashed lines) were counted as IENF (arrows), S100-positive Schwann cell processes co-localized with them (arrowheads). S100-positive Langerhans cells close to the epidermal-dermal border were excluded (asterisk). Scale bars = 50 µm

**Fig. 4:**
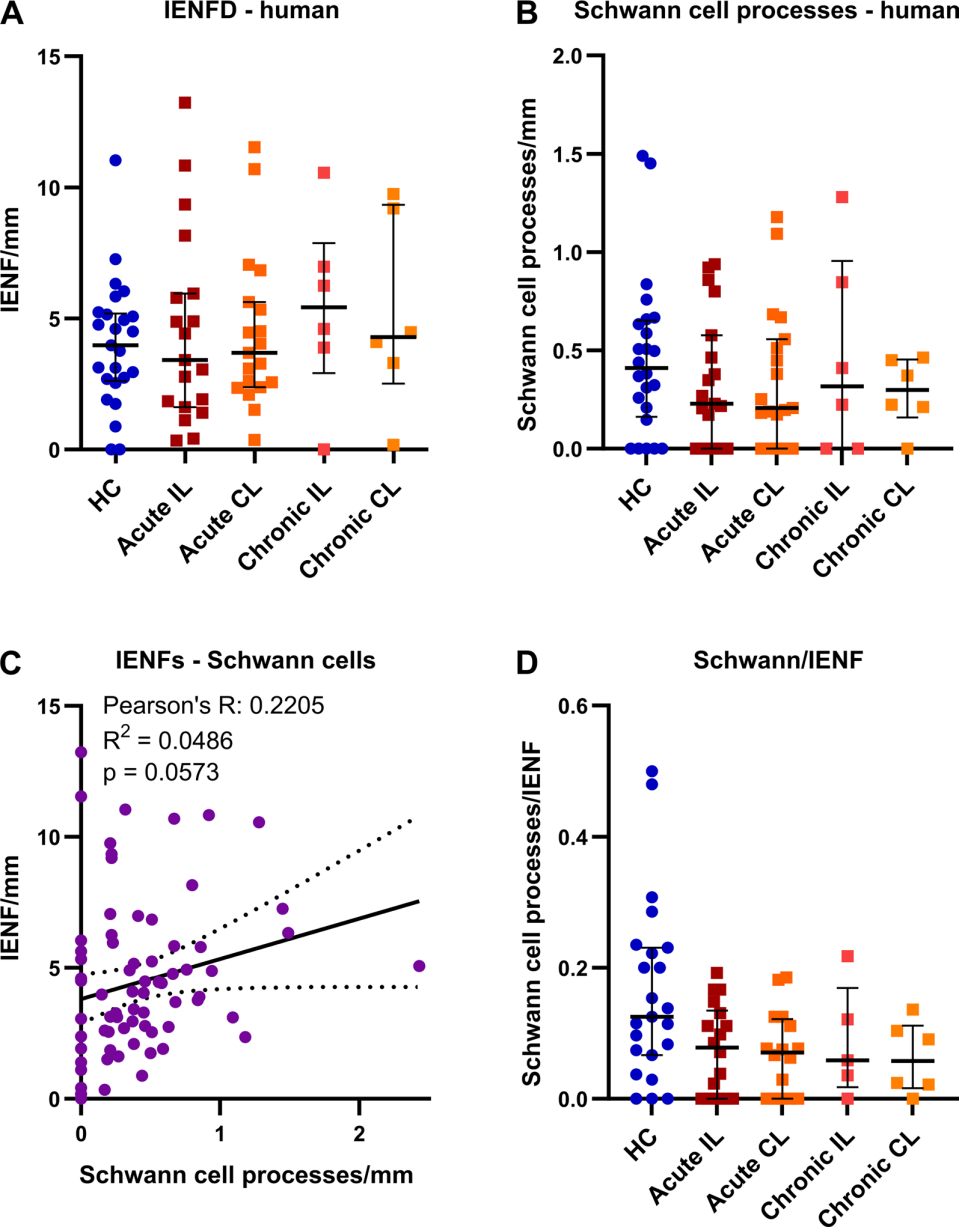
Preserved intraepidermal nerve fibre and Schwann cell innervation in CRPS affected skin. Skin sections from the index finger were immunolabelled to assess **A** intraepidermal nerve fibre density (IENFD, n_HC_ = 25, n_acute_ = 19, n_chronic_ = 6) and **B** intraepidermal Schwann cell process density in patients with acute and chronic CRPS and HC (n_HC_ = 24\1, n_acute_ = 19, n_chronic_ = 6). **C** Correlation of IENFD and Schwann cell process density in all skin samples (n = 75). **D** Ratio of Schwann cell processes and IENFs (n_HC_ = 25, n_acute IL_ = 18\1, n_acute CL_ = 16\3 n_chronic_ = 6). Data are presented as median ± interquartile range (Kruskal–Wallis and Dunn’s tests). CL: contralateral; HC: healthy controls; IL: ipsilateral

Representative images of IENFs with and without intraepidermal Schwann cell processes in human are shown in Fig. 3. In humans, the IENFDs were similar between the groups and all CRPS patients—acute or chronic—had IENFDs above the 5% percentile of healthy controls (HC, Fig. 4A). Acute CRPS type II patients—characterized by signs of nerve lesion—only showed a trend of decreased IENFD (Additional file 1: Fig. S1) which might be due to the small sample numbers for subgroup analysis. The density of Schwann cell processes was similar to HC in acute and chronic CRPS (Fig. 4B). Different to murine skin, Schwann cell processes did not correlate with the IENFD in human (Fig. 4C). To estimate if the CRPS patients had a higher ratio of unaccompanied nerve fibres, we determined the ratio of IENFs associated with Schwann cell processes. There was no loss of intraepidermal Schwann cells in relation to IENFs in acute or chronic CRPS patients compared to HC (Fig. 4D). Meissner corpuscle numbers were not changed in the acute CRPS cohort (Additional file 1: Fig. S2A, B). These results suggest that there is no initial loss of IENFs or intraepidermal Schwann cells.

### Accumulation of mast and Langerhans cells in acute, but not chronic CRPS

**Fig. 5:**
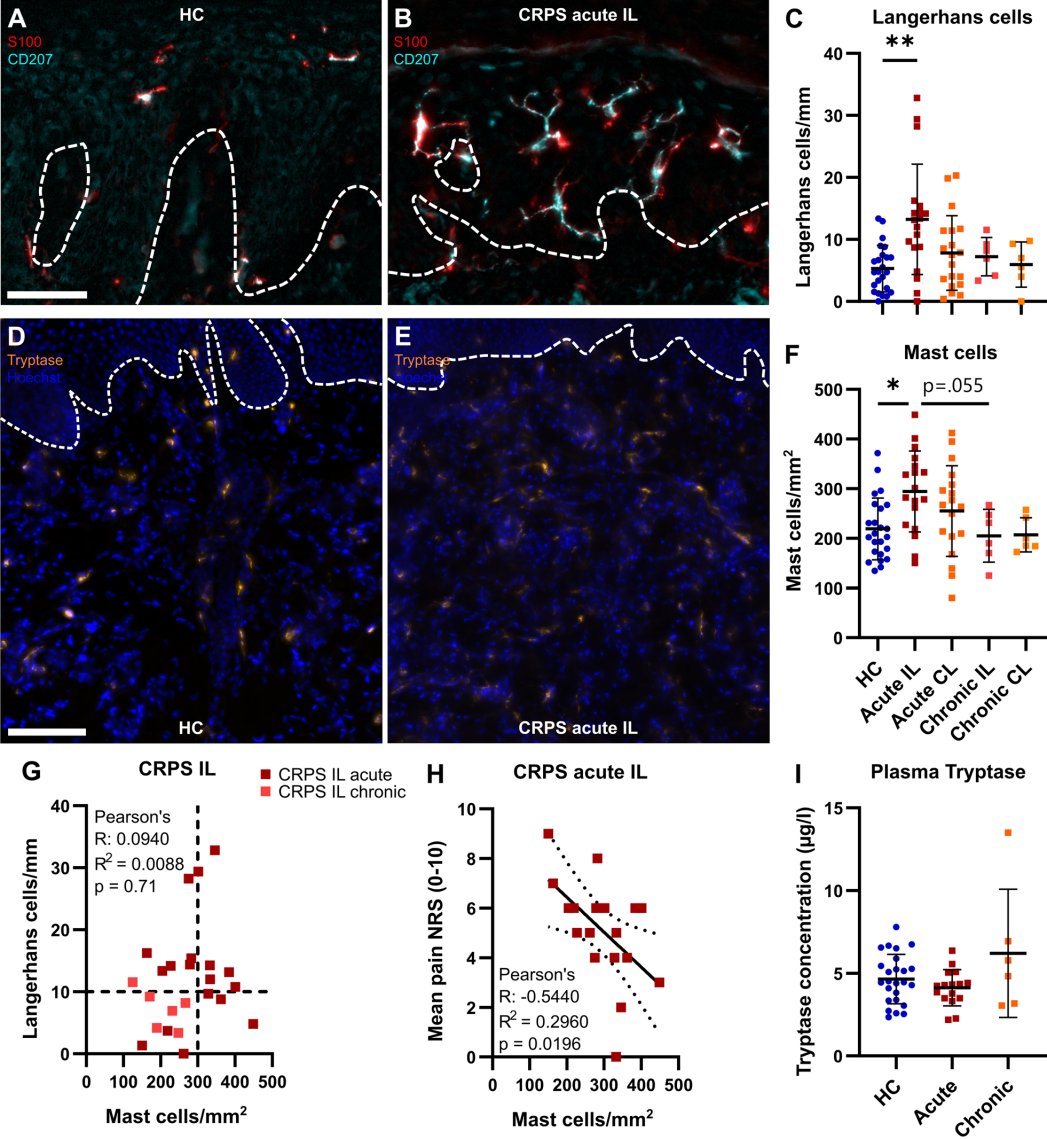
Prominent Langerhans and mast cell accumulation in CRPS affected finger of acute ipsilateral CRPS skin. Skin sections from finger biopsies were immunofluorescently labelled. **A**-**C** Representative images and quantification of Langerhans cells (S100^+^CD207^+^) in the epidermis are depicted. Dashed lines indicate the epidermal-dermal border (n_HC_ = 24\1, n_acute_ = 19, n_chronic_ = 6). Scale bar = 50 µm. **D**-**F** Representative images and quantification of mast cells (tryptase^+^) in the dermis but also close to the epidermal-dermal border (dashed lines). Nuclei were stained with Hoechst33342. Mast cells were quantified per area of dermis (n_HC_ = 24, n_acute_ = 18, n_chronic_ = 6). Scale bar = 100 µm. **G**, **H** Pearson correlation of Langerhans and mast cell densities or mast cell densities and mean numeric pain scale (NRS). Dashed lines indicate thresholds determined from data of HC group. **I** Tryptase concentrations were determined in plasma from patients with acute and chronic CRPS and compared with HCs (n_HC_ = 25, n_acute_ = 16\1, n_chronic_ = 6). Data are shown as mean ± SD; Welch’s ANOVA and Dunnett’s tests; *: p < 0.05; **: p < 0.01. CL: contralateral; HC: healthy controls; IL: ipsilateral

To characterize our CRPS patient cohorts regarding their immune response and its possible contribution to pain development and/or maintenance, we assessed the densities of Langerhans and mast cells. Langerhans cells are the major antigen-presenting cells in the skin and restricted to the epidermis. More S100^+^CD207^+^ Langerhans cells were identified in ipsilateral skin from acute CRPS compared with HC, but not in chronic CRPS (Fig. 5A–C). Mast cells are tissue-resident immune cells and—in the skin—reside in the dermis (Fig. 5D, E). The mast cell density was higher in acute IL CRPS skin compared to HC and chronic IL CRPS (Fig. 5F). Although both cell types showed higher abundance in acute IL CRPS skin, their sheer numbers were independent of each other (Fig. 5G). Interestingly, mast cell numbers in ipsilateral skin from acute CRPS correlated inversely with the self-reported mean NRS pain (Fig. 5H) but with none of the other clinical parameters (time since diagnosis, time since event, CSS, max NRS, temperature difference). To find out whether the increase of tryptase^+^ mast cells might be reflected in blood tryptase levels, fluoroenzyme immunoassay was performed. However, tryptase concentration in plasma was unaltered between groups (Fig. 5I). Langerhans cell density did not correlate with any clinical parameters. So, in acute CRPS in the affected limb, mast cell or Langerhans cell numbers are increased but normalize in the chronic/intermediate phase.

Complement component C5a poses another pro-inflammatory mediator with pro-nociceptive capacity in the skin [30–32]. In the tibia fracture and casting model of CRPS, C5a has been shown to be involved in the IgM-induced autoimmune cascade eliciting pain [33]. We therefore tested for changes in C5a immunoreactivity in CRPS affected human skin. Immunoreactivity was located predominantly in blood vessels. Although there were differences between biopsies regarding immunoreactivity and staining intensity, there were no apparent differences between HC and CRPS IL or CL. Furthermore, we observed that mast cells were often accumulated around C5a-positive structures (mainly blood vessels, Additional file 1: Fig. S3A). Intensity analysis did not reveal any differences between the groups (Additional file 1: Fig. S3B). Our results indicate that CRPS could be initially driven by different mechanisms mediated by either mast or Langerhans cells.

### No infiltration of cutaneous B and T cells in CRPS

**Fig. 6:**
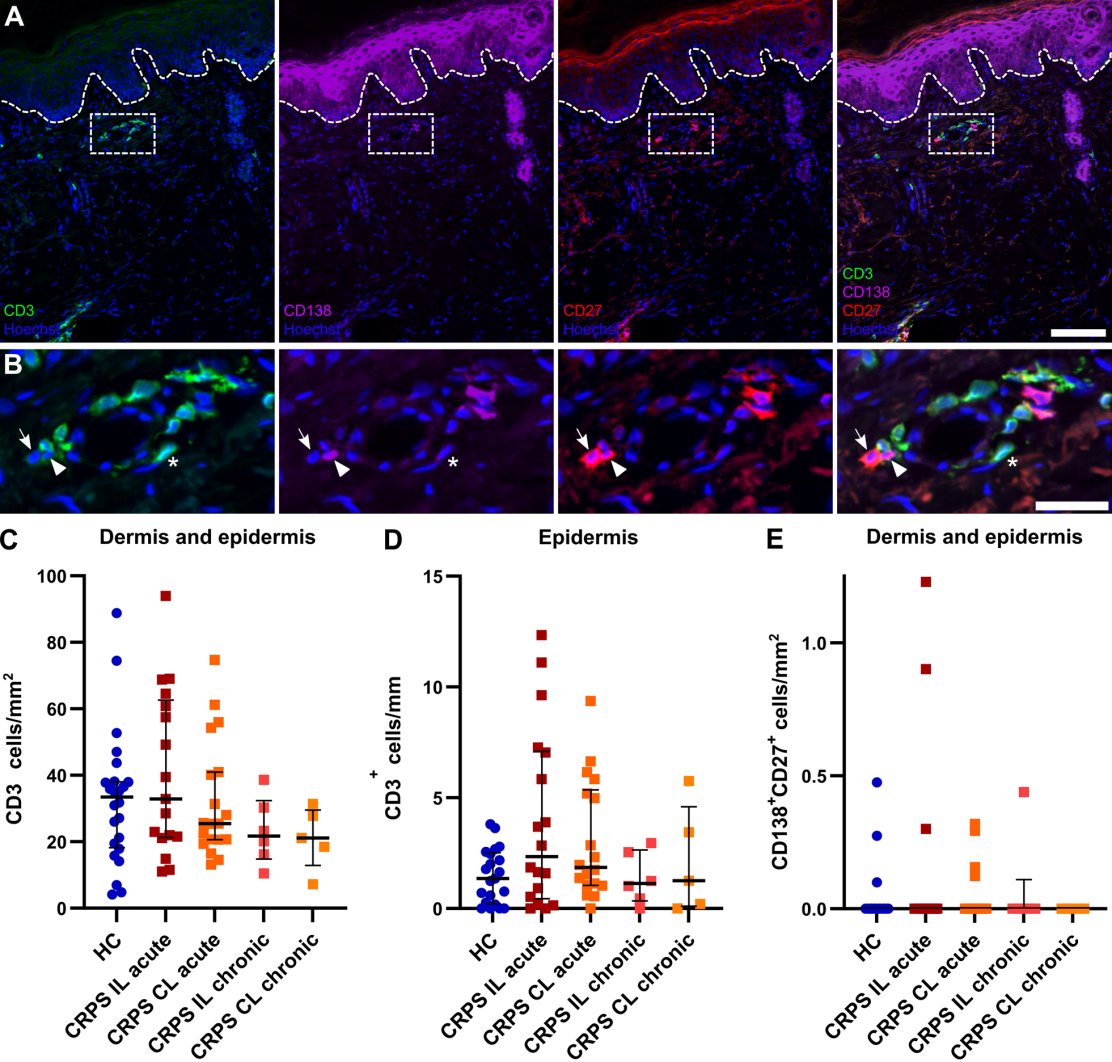
No apparent lymphocyte infiltration into skin from patients with acute or chronic CRPS. Skin biopsy of the index finger with** A** T cells (CD3^+^; asterisk) and plasma cells (CD27^+^CD138^+^; arrow and arrowhead). Scale bar = 100 µm. Dashed box indicates **B** the zoom-in area with T and plasma cells. Scale bar = 30 µm. **C**, **D** Densities of T cells in the dermis and epidermis of acute and chronic CRPS patients and HC are depicted. **E** Plasma cells (CD27^+^CD138^+^) were only detected in 7 samples; outliers were not excluded for plasma cells. Data are shown as median ± interquartile range; Kruskal–Wallis and Dunn's tests; **C** n_HC_ = 24, n_acute IL_ = 18\1, n_acute CL_ = 19, n_chronic IL/CL_ = 5/6; **D** n_HC_ = 21\3, n_acute IL_ = 19, n_acute CL_ = 18\1, n_chronic IL/CL_ = 5/6. CL: contralateral; HC: healthy controls; IL: ipsilateral

We next hypothesized that the observed increased Langerhans and mast cell numbers would lead to lymphocyte recruitment at the affected site. Autoimmune mechanisms have been described in early CRPS [10, 33, 34] and in other autoimmune-mediated skin conditions, such as psoriasis, T and B cells are involved and accumulate in the skin [35–38]. We quantified CD3^+^ T cells to assess possible cellular immune activation and CD27^+^CD138^+^ plasma cells—antibody-producing resident B cells—for humoral involvement, which might be reflected by lymphocyte infiltration (Fig. 6A, B). We assessed T cell densities in the whole skin section (dermis and epidermis) and the epidermis only, since skin-resident T cells predominantly persist in the epidermis [38]. Both T cell densities of the entire skin and the epidermal T cell density were similar in the acute and chronic CRPS groups compared with HC (Fig. 6C, D). Plasma cells were found only in 10 out of 72 samples but were not restricted to CRPS samples (Fig. 6E). Both CRPS cohorts showed no infiltration of CD3^+^ T cells or CD27^+^CD138^+^ plasma cells into the skin suggesting that tertiary lymphoid organ formation or obvious immune cell infiltration do not occur in the skin of acute CRPS patients. So, the innate immune system is activated while the adaptive is—at least when judged by number of cells—unaltered.

## Discussion

To elucidate CRPS aetiology, we systematically investigated various cell types potentially causing pain development and maintenance in the skin of acute and chronic CRPS patients. We found a non-simultaneous proliferation and/or immigration of Langerhans and mast cells in acute CRPS, but the glioneural complex, T and plasma cells were not affected. Furthermore, in our acute CRPS cohort, mast cell numbers were negatively associated with reported pain. Interestingly, this neuroimmune cell type profile in the skin from our relatively small chronic CRPS cohort did not show any changes. We confirmed the existence of cutaneous Schwann cells that accompany small nerve fibres across the epidermal-dermal border in human skin, however, the number was substantially lower than in mice.

To validate our counting method in the human skin, we first assessed nociceptive/intraepidermal Schwann cells in mice. CCI and sham mice served as models but had comparable IENFD. The regional differences in innervation in plantar mouse skin, high variability, or a short injury duration might have obscured a loss in IENFD [39]. Most importantly, we could detect many intraepidermal Schwann cell processes and their density correlated strongly with the IENFD, matching with the data and the reported interdependence of nociceptive Schwann cells and IENFs [22]. These results and our correlation analysis support the validity of our counting method.

Since CRPS patients suffer from neuropathic-like pain and IENF loss has been described [17, 20], we examined this new cell type in our cohorts. We defined S100^+^/CD207^−^ processes with PGP9.5^+^ nerve fibres crossing the dermal-epidermal border as intraepidermal Schwann cell processes. Cutaneous Schwann cells were documented in the leg from healthy humans—though without quantification [22]. In another study processes of Schwann cells accompanying IENFs were not detected in human hairy skin from the leg; instead subepidermal Schwann cell somata and their branches associated with IENFs in the dermis were quantified [40]. So, different skin areas might harbour different numbers of intraepidermal Schwann cells.

Acute and chronic CRPS patients had normal IENFD and similar numbers of associated Schwann cell processes accompanying IENFs through the dermal-epidermal border. This contradicts most studies of cutaneous nerve fibres in CRPS, but in these mainly patients with very long-standing CRPS were examined [17, 18, 20, 41]. *Rasmussen* and colleagues suggested—based on their results of 100% of their 8 patients with bilateral IENF loss—small fibre loss as a predisposing factor for CRPS [17]. In our acute CRPS type II subgroup, there was only a trend of fibre loss in the affected side compared with all other groups, including the unaffected side. This might be due to the small sample size or the early disease state. Increasing the sample size or analysis of chronic CRPS type II biopsies might reveal a more prominent fibre loss. Our findings could not confirm the hypothesis of reduced IENFD as a prerequisite and contributor to pain in CRPS. Furthermore, the demise of IENF or intraepidermal Schwann cells does not seem to be a rapid immune cell-driven mechanism: our small chronic CRPS cohort had normal IENFD with normal immune cell numbers.

The transient accumulation of mast and Langerhans cells in our acute CRPS cohort argues for immune activation with subsequent resolution—at least in cell number. These results are in accordance with previous studies [7, 14, 16]. The inflammatory nature of mast cells is well known: upon activation they secrete a wide range of pro-inflammatory mediators, such as prostaglandins, leukotrienes or cytokines, e.g. IL-6 and TNF-α [42]. Mast cells can directly mediate pain as demonstrated in the CRPS rat tibia fracture model [43]. In a postoperative pain model, skin mast cells alone induced pain by producing GTP hydrolase, the rate-limiting enzyme for tetrahydrobiopterin (BH4). Interestingly, this could be triggered by substance P [44], a neuropeptide involved in CRPS pathophysiology [43, 45, 46]. While increased mast cell counts can reflect activation and degranulation, the quantity and types of degranulated molecules remain unknown using our method. For instance, differences in tryptase levels from skin blister fluids were much more pronounced [14] than our mast cell counts comparing CRPS and HC. Both our CRPS cohorts had similar mean pain and CRPS severity, but elevated mast cell numbers were only present in the acute cohort and correlated negatively with pain intensity. This seems counterintuitive given their known pro-inflammatory effects. But anti-inflammatory functions of mast cells have been described: in the mouse model of severe contact hypersensitivity, dermal mast cells release IL-10 and counteract inflammatory symptoms and tissue pathology [47]. But in the milder model, mast cells do not release IL-10 [47]. Similarly, mast cells in CRPS might counterregulate inflammation and pain only in the acute state with increasing density. In the chronic CRPS cohort, the number of mast cells normalized. The remaining mast cells might still release granules and higher amounts of pro-nociceptive molecules despite their lower numbers. In skin blister fluid from chronic CRPS, cytokine levels remained elevated [8, 48] supporting this hypothesis. Another possibility for persistent pain in chronic CRPS might be central sensitization: neuron-mast cell crosstalk has been described in several contexts and might potentiate neurogenic inflammation and account for central sensitization [49]. In line with that, mast cell-targeting treatment has been proposed to alleviate inflammation and prevent central sensitization in CRPS [42]. Quantifying mast cells in acute CRPS might indicate if mast cell-specific treatment might be an option. Meticulous investigations of mast cells are needed to elucidate their pathophysiological role and possible treatment options for CRPS.

The number of Langerhans cells was increased independent of mast cell numbers. An elevation of Langerhans cells in CRPS has been described before in both acute and chronic CRPS [15, 50], but not in all studies [16]. Langerhans cells were associated with burning pain [51]. However in the preclinical CRPS tibia fracture model, Langerhans cell depletion did not affect mechanical thresholds [15]. Langerhans cells can be divided into two steady-state types transferring innate immunity and antigen presentation while the two inflammatory types mediate leukocyte activation and adaptive immunity [52]. So, a detailed analysis is necessary to estimate inflammatory and pro-algesic processes in the skin of the two independent immunological drivers for CRPS development: mast and Langerhans cells.

We hypothesized that Langerhans cells might recruit or activate lymphocytes in the affected skin in CRPS. However, we observed no changes in CD3^+^ T cells and CD27^+^CD138^+^ plasma cells in our skin biopsies. Previous studies have described autoimmune involvement in CRPS [10, 34, 53–55]. Although mostly autoantibodies from serum were investigated, cloned T cells from affected compared with unaffected CRPS skin showed a trend to increased IL-13 secretion in a small cohort [16]. In long-standing CRPS, a phenotype shift of circulating T cells has been demonstrated [34]. After recruitment and infiltration into the skin, T cells can persist in the epidermis as skin-resident T cells and be rapidly reactivated by a later stimulus [38]. In psoriasis and other dermal diseases, T cell infiltration is substantial [56]. However, our T cell numbers in the epidermis or in the entire skin were completely normal, implying that further analysis of T cell phenotypes (e. g., by CD4 and CD8) seemed unpromising. B cell accumulation in diseased skin has also been demonstrated [36, 57]. There is evidence for autoantibody involvement in at least early CRPS [10] but we barely found antibody-producing plasma cells in any of the skin samples. We did not check for autoimmune involvement in blood from our CRPS cohorts. Future studies should however have a double approach and prior test for autoantibodies or shifted T cell subsets in blood. There might still be cutaneous changes specific to autoimmune involvement indecipherable to our blunt method.

This interim study is limited by the number of samples, especially of the chronic CRPS cohort, since only patients with affection of the upper extremities were included. Furthermore, the biopsies from the chronic cohort were not follow-ups from the acute patients. Drawing conclusion from follow-up biopsies of the same CRPS patients would better reflect the time course of the disease. Due to the small cohorts, it is hard to make a statement about the infiltration or proliferation of immune cells but our data and their interpretations seem plausible. Additionally, we only counted the investigated cell types and did not perform other analyses regarding activation or phenotype status. More sophisticated examinations would require other methods such as single-cell RNA sequencing. The strengths of this study are the control tissues, since we analysed both healthy controls and unaffected contralateral biopsies from the same biopsy site. Our study participants received thorough clinical investigation and characterization using an array of questionnaires and thorough clinical assessment. Additionally, we investigated multiple parameters and cell types within the same patient cohort presenting a clearer picture of changed and constant parameters.

## Clinical implications and conclusion

In our study, we observed enhanced immune sentinel cell activation, gauged by Langerhans and mast cell accumulation in the acute cohort. Thus, anti-inflammatory therapy, e.g. a steroid pulse, would be recommended across all acute CRPS groups, but not all individual patients. Furthermore, although CRPS patients report sensory plus symptoms resembling neuropathic pain and respond to anti-neuropathic treatment, we did not find typical patterns of small nerve fibre affection. Even in CRPS type II, there was not a clear loss of IENFs or intraepidermal Schwann cells. Detailed functional analyses are necessary to investigate cutaneous cells more thoroughly. Full transcriptomic or proteomic analyses will help to understand the pathophysiology and provide specific treatment options.

What triggers this reaction and how and why this elicits pain remains unanswered. Inflammatory, pro-algesic molecules might play a role, supported by the classical view of the “vicious circle”: aberrant release of neuropeptides from nerve fibres activates immune cells, which in turn release pro-algesic molecules leading to further aberrant firing [7]. Because CRPS is not a skin disease, the central somatosensory system should be investigated additionally, e.g. spinal cord and/or brain, especially in the early disease phase. In the future, detailed clinical and molecular phenotyping will guide tailored treatment depending on the CRPS aetiology.

### Supplementary Information

**Additional file 1: Figure S1.** Intraepidermal nerve fibre and Schwann cell densities in type I and II CRPS. **(A) **Intraepidermal nerve fibre density (IENFD) in acute CRPS type I and II patients compared with HC. **(B)** The density of Schwann cell processes was quantified. **(C)** Ratio of Schwann cell process accompanied IENFs are depicted. Data are presented as median and interquartile range (Kruskal-Wallis and Dunn's tests; n_HC _= 25, n_acute ipsi type I _= 11, n_acute ipsi type II _= 7\1). CL: contralateral; HC: healthy controls; IL: ipsilateral. **Figure S2.** Meissner corpuscle density in skin from patients with CRPS and healthy controls **(A) **Representative image of a Meissner corpuscle in a collagen IV labelled papilla. **(B)** Quantification of Meissner corpuscle density. Data are presented as median and interquartile range (Kruskal-Wallis and Dunn's tests; n_HC _= 25, n_acute-IL _= 18\1, n_acute _= 19, n_chronic _= 6). CL: contralateral; CRPS: complex regional pain syndrome; HC: healthy controls; IL: ipsilateral. **Figure S3.** Similar C5a mean intensity in the dermis from CRPS patients compared with healthy controls. **(A) **Representative image of C5a stainings. Scale bar = 200 µm. **(B)** Quantification of C5a mean immunofluorescence of z-stack maximum projections. Data are presented as median and interquartile range (Welch’s ANOVA and Dunnett’s tests; n_HC _= 21\4, n_acute _= 18, n_chronic _= 6). CL: contralateral; CRPS: complex regional pain syndrome; HC: healthy controls; IL: ipsilateral. **Table S1.** Detailed demographic and clinical data of each CRPS patient.

## Data Availability

The datasets used and/or analysed during the current study are available from the corresponding author on reasonable request.

## Abbreviations

C5a: Complement component 5a
CCI: Chronic constriction injury
CD: Cluster of differentiation
CL: Contralateral
CRPS: Complex regional pain syndrome
DFNS: *Deutscher Forschungsverbund Neuropathischer Schmerz* (German Research Network on Neuropathic Pain)
HC: Healthy control
IENF: Intraepidermal nerve fibre
IENFD: Intraepidermal nerve fibre density
IL: Ipsilateral
IL-6: Interleukin-6
NRS: Numeric rating scale
ROI: Region of interest
TNF-α: Tumour necrosis factor α
