# Supplementary material for: Transient immune activation without loss of intraepidermal innervation and associated Schwann cells in patients with complex regional pain syndrome
Source: J Neuroinflammation. 2024 Jan 17;21:23. doi: 10.1186/s12974-023-02969-6 (PMC10792943; doi:10.1186/s12974-023-02969-6)
Supplement: Supplementary file 1 — Additional file 1: Figure S1. Intraepidermal nerve fibre and Schwann cell densities in type I and II CRPS. (A) Intraepidermal nerve fibre density (IENFD) in acute CRPS type I and II patients compared with HC. (B) The density of Schwann cell processes was quantified. (C) Ratio of Schwann cell process accompanied IENFs are depicted. Data are presented as median and interquartile range (Kruskal-Wallis and Dunn's tests; nHC = 25, nacute ipsi type I = 11, nacute ipsi type II = 7\1). CL: contralateral; HC: healthy controls; IL: ipsilateral. Figure S2. Meissner corpuscle density in skin from patients with CRPS and healthy controls (A) Representative image of a Meissner corpuscle in a collagen IV labelled papilla. (B) Quantification of Meissner corpuscle density. Data are presented as median and interquartile range (Kruskal-Wallis and Dunn's tests; nHC = 25, nacute-IL = 18\1, nacute = 19, nchronic = 6). CL: contralateral; CRPS: complex regional pain syndrome; HC: healthy controls; IL: ipsilateral. Figure S3. Similar C5a mean intensity in the dermis from CRPS patients compared with healthy controls. (A) Representative image of C5a stainings. Scale bar = 200 µm. (B) Quantification of C5a mean immunofluorescence of z-stack maximum projections. Data are presented as median and interquartile range (Welch’s ANOVA and Dunnett’s tests; nHC = 21\4, nacute = 18, nchronic = 6). CL: contralateral; CRPS: complex regional pain syndrome; HC: healthy controls; IL: ipsilateral. Table S1. Detailed demographic and clinical data of each CRPS patient. [file 12974_2023_2969_MOESM1_ESM.docx]

**Additional files:**


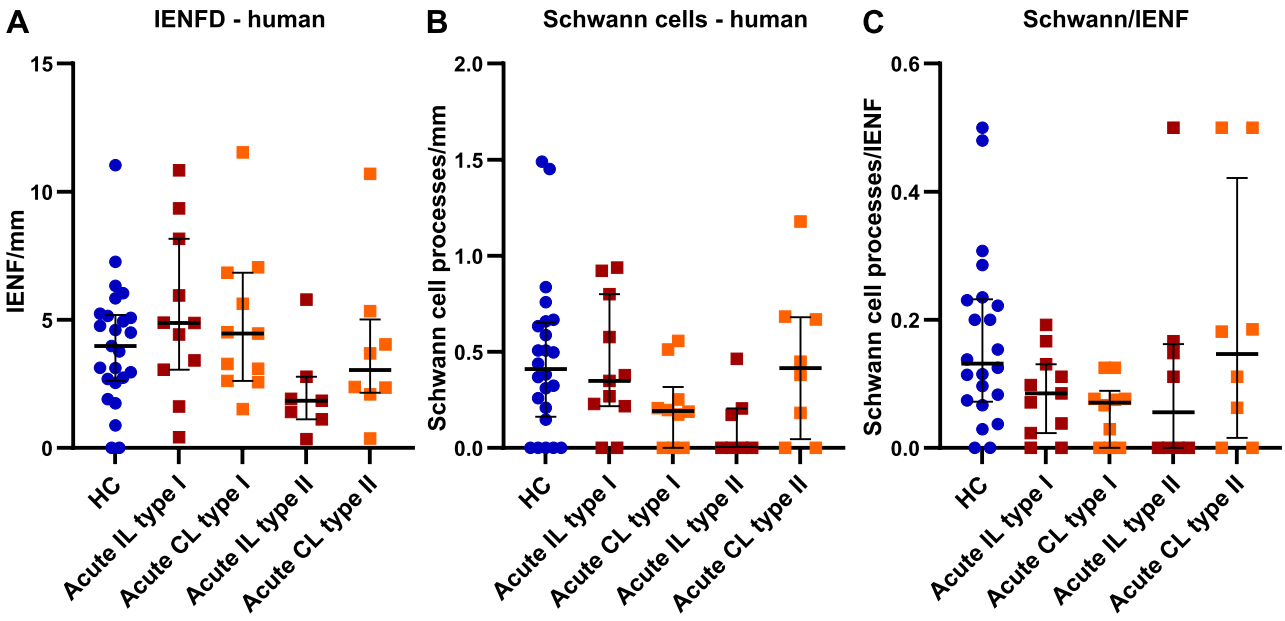


**Figure S1: Intraepidermal nerve fibre and nociceptive Schwann cell densities in type I and II CRPS. (A)** Intraepidermal nerve fibre density (IENFD) in acute CRPS type I and II patients compared with HC. **(B)** The density of Schwann cell processes was quantified. **(C)** Ratio of Schwann cell process accompanied IENFs are depicted. Data are presented as median and interquartile range (Kruskal-Wallis and Dunn's tests; n_HC_= 25, n_acute ipsi type I_= 11, n_acute ipsi type II_= 7\1). CL: contralateral; HC: healthy controls; IL: ipsilateral.


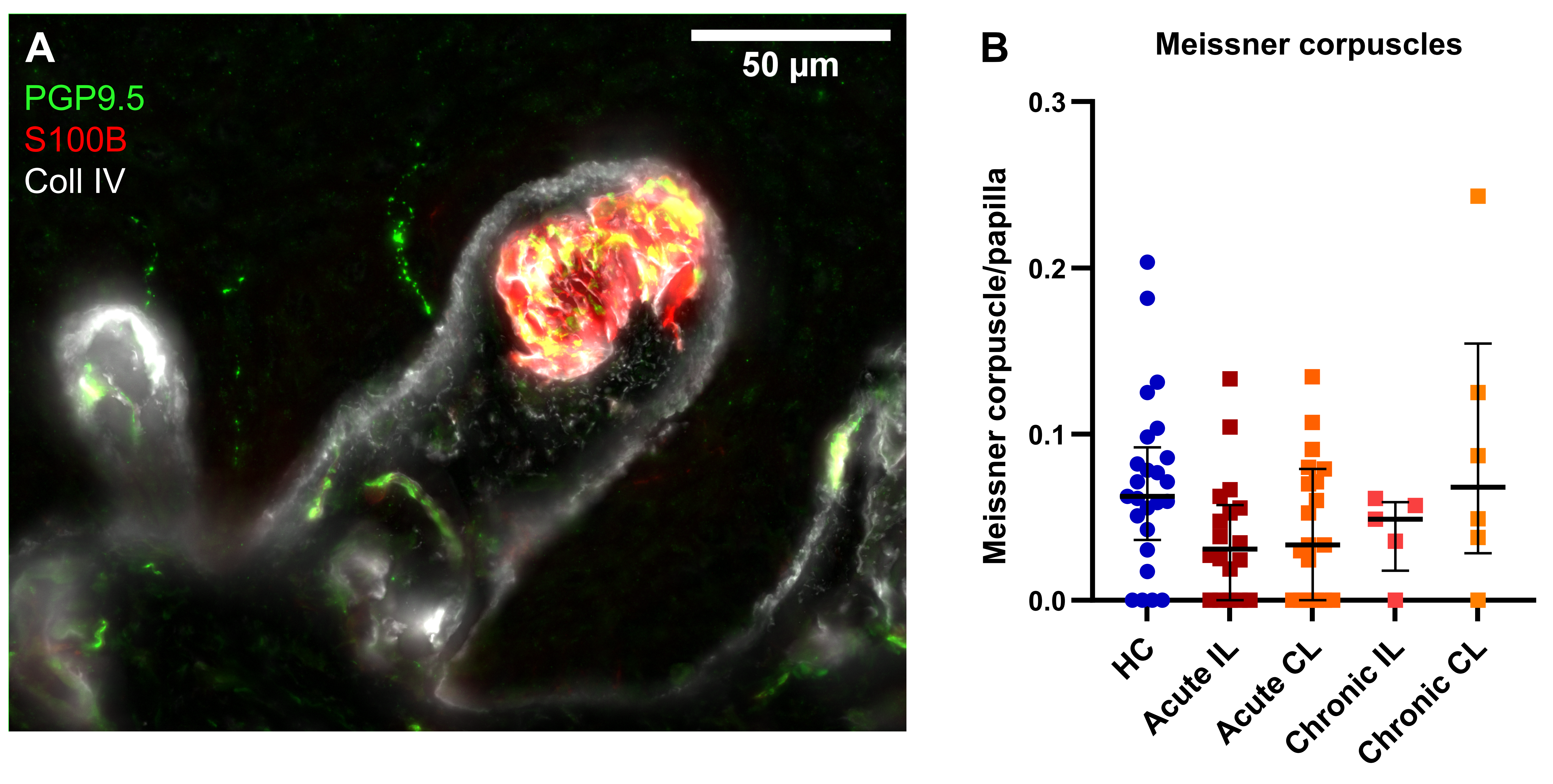


**Figure S2: Meissner corpuscle density in skin from patients with CRPS and healthy controls (A)** Representative image of a Meissner corpuscle in a collagen IV labelled papilla. **(B)** Quantification of Meissner corpuscle density. Data are presented as median and interquartile range (Kruskal-Wallis and Dunn's tests; n_HC_= 25, n_acute-IL_= 18\1, n_acute_= 19, n_chronic_= 6). CL: contralateral; CRPS: complex regional pain syndrome; HC: healthy controls; IL: ipsilateral.


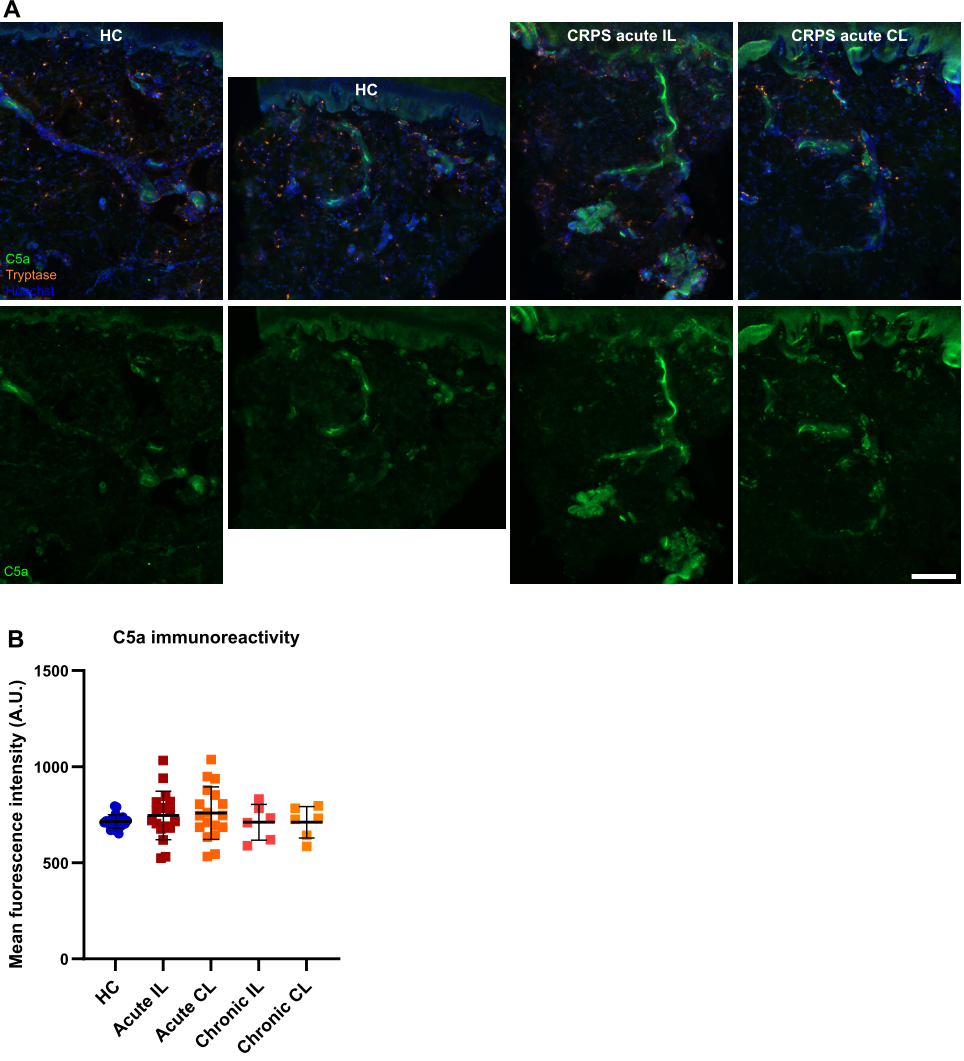


**Figure S3: Similar C5a mean intensity in the dermis from CRPS patients compared with healthy controls. (A)** Representative image of C5a stainings. Scale bar = 200 µm. **(B)** Quantification of C5a mean immunofluorescence of z-stack maximum projections. Data are presented as median and interquartile range (Welch’s ANOVA and Dunnett’s tests; n_HC_= 21\4, n_acute_= 18, n_chronic_= 6). CL: contralateral; CRPS: complex regional pain syndrome; HC: healthy controls; IL: ipsilateral.

**Table S1. Detailed demographic and clinical data of each CRPS patient.**

| **Age (years)** | **Sex** | **CRPS I/II** | **Time since diagnosis (months)** | **Time since event (months)** | **Mean pain (NRS)** | **Oede-ma** | **Allo-**  **dynia** | **Hyper-**  **algesia** | **Skin temp (Δ°C)** | **CSS** | **Initially warm** | **Type of trigger** | **Location** |
| --- | --- | --- | --- | --- | --- | --- | --- | --- | --- | --- | --- | --- | --- |
| 54 | f | I | 3 | 1 | 4 | + | - | + | -2.7 | 12 | + | Fracture + Surgery | Finger |
| 71 | f | I | 4 | 1 | 2 | + | - | + | -1.8 | 13 | + | Fracture + Surgery | Forearm |
| 53 | f | I | 4 | 0 | 0 | + | - | + | -0.9 | 10 | + | Fracture + Surgery | Forearm |
| 53 | f | I | 3 | 1 | 3 | + | - | - | -0.7 | 10 | + | Fracture + Surgery | Forearm |
| 53 | f | I | 11 | 11 | 5 | - | + | + | -0.6 | 10 | + | CTS surgery | Hand |
| 70 | f | I | 7 | 2 | 6 | + | - | - | -0.3 | 7 | n. d. | Fracture + Surgery | Forearm |
| 40 | f | I | 10 | 1 | 6 | - | - | - | -0.1 | 9 | + | Blunt trauma | Forearm |
| 53 | f | I | 6 | 1 | 9 | - | + | - | 0.5 | 9 | n. d. | Surgery | Upper arm |
| 36 | f | I | 10 | 2 | 6 | + | + | + | 1 | 13 | - | Sharp trauma | Finger |
| 57 | m | I | 3 | 1 | 4 | + | - | + | 1.4 | 12 | + | Sharp trauma + Surgery | Hand |
| 56 | f | I | 8 | 2 | 6 | + | + | - | 1.4 | 10 | + | Fracture + Surgery | Hand |
| 54 | m | II | 3 | 1 | 7 | + | - | + | -2.3 | 12 | n. d. | Blunt trauma | Forearm |
| 61 | f | II | 6 | 6 | 6 | + | - | - | -1.8 | 9 | + | Surgery | Finger |
| 51 | m | II | 2 | 0 | 5 | + | - | + | -1.7 | 13 | + | CTS surgery | Hand |
| 53 | f | II | 5 | 2 | 6 | - | + | + | -0.8 | 10 | + | Surgery | Hand |
| 55 | f | II | 4 | 1 | 8 | + | + | + | -0.1 | 8 | n. d. | CTS surgery | Hand |
| 66 | m | II | 4 | 3 | 8 | + | - | - | 0.1 | 8 | n. d. | Blunt trauma | Upper arm |
| 55 | m | II | 2 | 1 | 5 | + | - | - | 0.7 | 9 | n. d. | Fracture + Surgery | Forearm |
| 40 | f | II | 3 | 2 | 4 | + | + | - | 2.9 | 13 | + | Blunt trauma | Forearm |
| 59 | f | I | 14 | 5 | 6 | + | - | - | -0.5 | 9 | + | Fracture + Surgery | Forearm |
| 31 | f | I | 24 | 21 | 6 | - | + | + | 0.7 | 14 | - | Fracture + Surgery | Forearm |
| 31 | f | I | 20 | 1 | 4 | - | - | + | 0.8 | 11 | - | Surgery | Forearm |
| 43 | f | I | 14 | 1 | 1 | + | - | + | 1 | 11 | + | Sharp trauma | Hand |
| 25 | f | I | 25 | 1 | 7 | + | - | - | 1.8 | 11 | n. d. | Sharp trauma | Hand |
| 51 | f | II | 17 | 15 | 6 | + | - | + | -0.6 | 8 | + | CTS surgery | Hand |

CTS: carpal tunnel syndrome; f: female; m: male; n. d.: not determined/known; NRS: numeric rating scale.
